# Nitrite is a cGMP generator in isolated platelets

**DOI:** 10.1186/2050-6511-16-S1-A65

**Published:** 2015-09-02

**Authors:** Alessandra Borgognone, Thomas Loka, Myriam Chimen, Ed Rainger, Martin Feelisch, Steve P Watson, Michael P Frenneaux, Melanie Madhani

**Affiliations:** 1 Centre for Cardiovascular Sciences, University of Birmingham, Birmingham, UK; 2 Norwich Medical School, University of East Anglia, Norwich, UK; 3 Clinical and Experimental Sciences, Faculty of Medicine, University of Southampton, Southampton, UK

## 

cGMP is generated in blood platelets following activation of soluble guanylate cyclase (sGC) by nitric oxide (NO). Once synthesised, cGMP activates cGMP-dependent protein kinase (PKG) and causes an increase in intracellular cAMP concentration by inhibiting phosphodiesterase 3 (PDE3), responsible for its degradation. PKG and the cAMP-dependent protein kinase (PKA) phosphorylate a number of substrates with inhibitory effects on platelet function [1]. While NO is mainly generated by endothelial NO synthase (eNOS) in the endothelium, reduction of nitrite constitutes an alternative vascular source of NO during hypoxic and acidic conditions. Nitrite originates from reduction of dietary nitrate, found in foods such as beetroot and green leafy vegetables. Nitrite in the circulation can be converted to NO by several nitrite reductases in blood and tissues [2], and triggers vasodilatation [3] thus lowering blood pressure [4]. It has also been demonstrated that when eNOS activity is impaired, NO generated through the nitrate/nitrite pathway exerts negative effects on platelet function [5].

Whilst deoxyhaemoglobin is a candidate nitrite-reductase in blood [6], we aimed to characterise the effects of nitrite in isolated platelets. As such, we used a preparation of washed platelets and supraphysiological concentrations of nitrite *in vitro* and characterised nitrite-dependent cGMP generation and inhibition of platelet function.

Our data show that nitrite at high concentrations (1mM) is a powerful generator of cGMP in platelets in the absence of extracellular reductases. While cGMP accumulation is inhibited by sGC inhibitor ODQ, NO scavengers have a partial effect. This seems to indicate that nitrite acts through both NO-dependent and independent mechanisms. Accordingly, phosphorylation of the the cAMP and cGMP-dependent substrate VASP is strongly increased and, similarly to cGMP generation, is completely dependent on sGC activity but not NO. The increase of cGMP induced by high concentrations of nitrite inhibits platelet function as measured by platelet aggregation and secretion. However, whilst being dependent on sGC activity, the effect of nitrite on aggregation is not dependent on NO.generation. Lower concentrations of nitrite synergise with inhibition of PDEs, in particular PDE5 responsible for cGMP degradation, to trigger detectable VASP phosphorylation and inhibition of aggregation.

Nitrite triggers generation of cGMP in platelets and exerts inhibitory effects independently of extracellular reductases and other blood cells. Nitrite acts partly through reduction to NO and partly through an uncharacterised direct effect on sGC.
